# Extracranial Vertebral Artery Dissecting Aneurysm Presenting as Vertebrobasilar Stroke in a Young Adult: Case Report of Flow-Diverter Stenting

**DOI:** 10.3390/neurolint17110187

**Published:** 2025-11-18

**Authors:** Maria Angelica-Coronel, Melissa Luque-Llano, Narledis Nuñez-Bravo, Carlos Rebolledo, Ernesto Barceló-Martínez

**Affiliations:** 1 Facultad de Ciencias de la Salud, Universidad Simón Bolívar, Barranquilla 080001, Colombia; melissa.luque@unisimon.edu.co (M.L.-L.); narledis.nunez@unisimon.edu.co (N.N.-B.); 2 Clinica Iberoamerica, Barranquilla 080001, Colombia; carlos.rebolledo@unisimon.edu.co (C.R.); erbarcelo@yahoo.com (E.B.-M.)

**Keywords:** vertebral artery, aneurysm, stroke, young adult, stents, dissection, vertigo, endovascular procedures, case report

## Abstract

**Background**: Extracranial vertebral artery aneurysms (EVAAs) are exceptionally rare vascular lesions and an uncommon cause of posterior circulation stroke. Their diagnosis is often delayed due to nonspecific symptoms, yet prompt recognition is essential to guide management. **Objective**: This study aimed to report a rare case of an extracranial vertebral artery dissecting aneurysm presenting as a posterior circulation stroke in a young adult, successfully managed with flow-diverter stenting. **Clinical Case**: A 33-year-old woman presented with sudden-onset dysarthria, vertigo, nausea, and vomiting. Brain magnetic resonance imaging revealed infarcts in the left occipital lobe, cerebellar peduncle, and both cerebellar hemispheres. Computed tomography angiography (CTA) demonstrated a fusiform aneurysm in the V2 segment of the left vertebral artery, and digital subtraction angiography (DSA) confirmed a dissecting aneurysm. The patient was successfully treated with a flow-diverting stent and remained stable at 6 months’ follow-up with mRS 1. **Results**: EVAA are uncommon but can manifest as posterior circulation ischemic events in young patients. Endovascular treatment with flow-diverting stents has been reported as a feasible option in selected cases, although evidence remains limited to case reports and small series. **Conclusions**: This case underscores the importance of considering rare yet potentially treatable etiologies of vertebrobasilar stroke in young patients and highlights the value of a multidisciplinary approach to management.

## 1. Introduction

The vertebrobasilar circulation accounts for approximately 25% of ischemic strokes, most of which are due to vertebral artery (VA) pathology, including atherosclerosis, fibromuscular dysplasia, trauma, or dissection, and less frequently connective tissue disorders [1]. Although aneurysms are recognized as a cause of posterior circulation stroke, vertebral artery aneurysms are exceedingly uncommon and typically located intracranially. Extracranial vertebral artery aneurysms (EVAAs) represent an exceptionally rare entity, accounting for <1% of all vertebral artery aneurysms. To date, only isolated case reports and very small series have been published, and therefore, no precise incidence or prevalence can be established [1,2].

Differential diagnosis should include not only arterial dissection but also mass lesions in the parapharyngeal or cervical region, such as parapharyngeal space tumors, branchial cysts, or inflammatory masses, which can mimic vascular lesions on clinical exam or non-contrast imaging [3].

Clinical manifestations range from compressive symptoms such as neck pain, dysphagia, and radiculopathy, to ischemic presentations including vertigo, diplopia, dysarthria, limb weakness, and ataxia [4].

The advent and refinement of advanced imaging modalities have substantially improved the diagnostic accuracy for EVAA. Computed tomography angiography (CTA) offers rapid, high-resolution visualization of vascular anatomy and bony structures but may be limited by bone overlap and radiation exposure [5]. Digital subtraction angiography (DSA) remains the gold standard for detailed luminal assessment and therapeutic planning, allowing hemodynamic evaluation and immediate intervention [6]. Magnetic resonance angiography (MRA), especially fat-suppressed T1 sequences, provides non-invasive visualization of both vessel lumen and wall pathology, including intramural hematomas, making it particularly valuable for differentiating aneurysm from dissection [7].

Here, we report a rare case of an extracranial vertebral artery dissecting aneurysm in a young adult, successfully treated with a flow-diverting stent. This case contributes to the limited literature and highlights the role of reconstructive endovascular strategies in selected patients.

## 2. Case Description

A 33-year-old right-handed woman presented to the emergency department with a three-day history of sudden-onset dysarthria, vertigo, nausea, and vomiting. The patient denied headache, diplopia, neck pain, sensory disturbances, limb weakness, seizures, or recent head or cervical trauma. She reported no recent infections, no contraceptive use, and no family history of cardiovascular or cerebrovascular disease. Her past medical history included a right salpingectomy due to ectopic pregnancy, a surgically treated right tibia and fibula fracture, and episodic migraine without aura. She was not taking any regular medications, did not smoke, and reported only occasional alcohol consumption. A detailed drug history, including illicit substances and over-the-counter medications, was specifically investigated and denied. There was no family history of cerebrovascular disease or aneurysms. No relevant psychosocial history was reported.

On admission, she was alert and oriented, with a Glasgow Coma Scale score of 15 [8]. Vital signs were stable: blood pressure 137/87 mmHg, heart rate 76 bpm, respiratory rate 18 breaths/min, oxygen saturation 98% on room air, and temperature 36.7 °C. Cardiac and pulmonary examinations were normal. Neurological examination revealed dysarthric speech, bilateral dysmetria, and dysdiadochokinesia, more pronounced on the right side, with truncal instability on tandem gait. Cranial nerves were intact, and there were no motor deficits, sensory disturbances, visual field defects, or meningeal signs. Deep tendon reflexes were symmetrical, and plantar responses were flexor bilaterally. The National Institutes of Health Stroke Scale (NIHSS) score [9] was 3 points: 1 point for dysarthria and 2 points for ataxia affecting both limbs. No cervical bruit or tenderness was identified.

Laboratory investigations, including complete blood count, electrolytes, renal and liver function, coagulation profile, thyroid function tests, inflammatory markers (C-reactive protein and erythrocyte sedimentation rate), autoimmune panel, thrombophilia screen, connective-tissue disorder evaluation, and pregnancy test, were within normal ranges or yielded negative results. Lipid profile showed LDL cholesterol of 118 mg/dL and HDL cholesterol of 56 mg/dL. ECG demonstrated sinus rhythm without conduction abnormalities. Transthoracic echocardiography revealed normal biventricular function, LVEF 54%, no valvular disease, and no patent foramen ovale. Twenty-four-hour Holter monitoring did not reveal arrhythmias.

**Figure 1:**
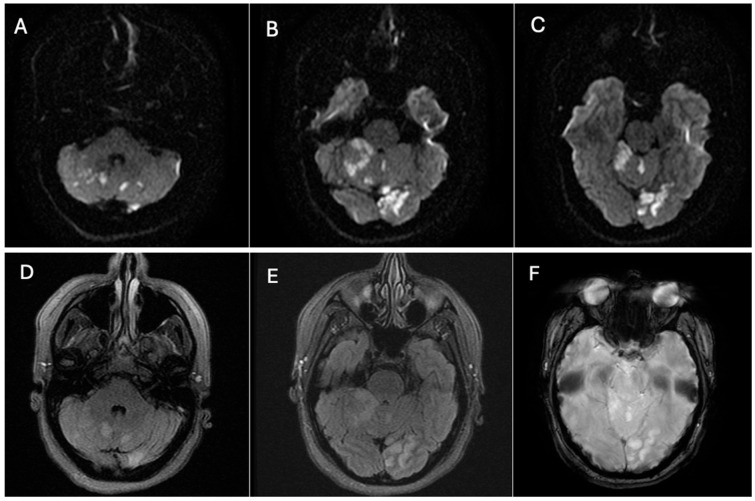
Brain magnetic resonance imaging (MRI): (**A**–**C**) Axial diffusion-weighted imaging (DWI) sequence (TR/TE = 3600/90 ms), showing multiple focal lesions in the left occipital lobe, left cerebellar peduncle, and both cerebellar hemispheres, more prominent on the right side. These lesions demonstrate marked diffusion restriction, consistent with acute embolic infarction. (**D**,**E**) Axial T2-weighted FLAIR sequence (TR/TE = 9000/120 ms), showing corresponding hyperintense signal abnormalities. (**F**) Axial gradient-echo T2* sequence (TR/TE = 700/20 ms), demonstrating no evidence of intracranial hemorrhage. Source: patient’s medical record, with prior authorization.

Neuroimaging was performed immediately. Non-contrast head CT revealed hypoattenuation in the parasagittal left occipital lobe, consistent with acute infarction. MRI of the brain confirmed acute ischemic lesions, showing multiple focal hyperintense lesions on T2/FLAIR with restricted diffusion involving the left occipital lobe, the left cerebellar peduncle, and both cerebellar hemispheres, more extensive on the right side, consistent with an embolic pattern of infarction (Figure 1).

**Figure 2:**
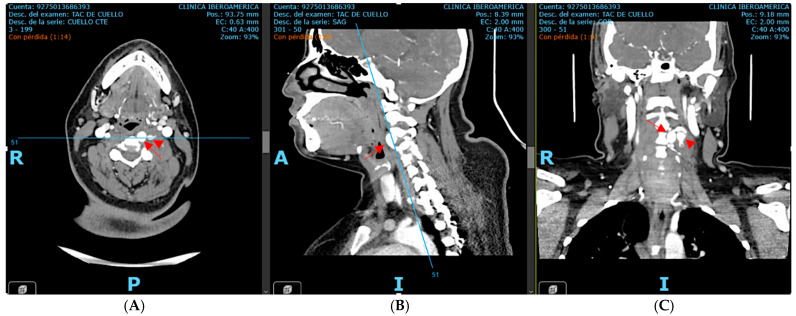
Contrast-enhanced CT angiography of the neck and cerebral vessels, axial view (**A**), sagittal view (**B**), and coronal view (**C**), performed after intravenous administration of 100 mL of non-ionic iodinated contrast medium (Iohexol, Omnipaque^®^ 300 mg I/mL, injection rate 3–4 mL/s). Image acquisition was performed during the arterial phase using a bolus-tracking technique (approximately 20–25 s after injection, triggered when the contrast density in the aortic arch reached ≥120 Hounsfield units). The study demonstrates a tortuous left vertebral artery with fusiform aneurysmal dilation of the V2 segment, measuring approximately 10 × 14 mm. A focal mural defect is identified, with active contrast extravasation into a contained perivertebral hypodense collection (34 × 24 mm). The aneurysmal dilation is marked by the red arrow, while the mural defect is indicated by the arrowhead. Source: patient’s medical record, published with prior authorization.

Subsequent computed tomography angiography (CTA) of the neck and cerebral vessels demonstrated a tortuous left vertebral artery with a fusiform aneurysm in the V2 segment measuring 10 × 14 mm, with an associated mural defect and active contrast extravasation into a perivertebral collection of 34 × 24 mm (Figure 2).

**Figure 3:**
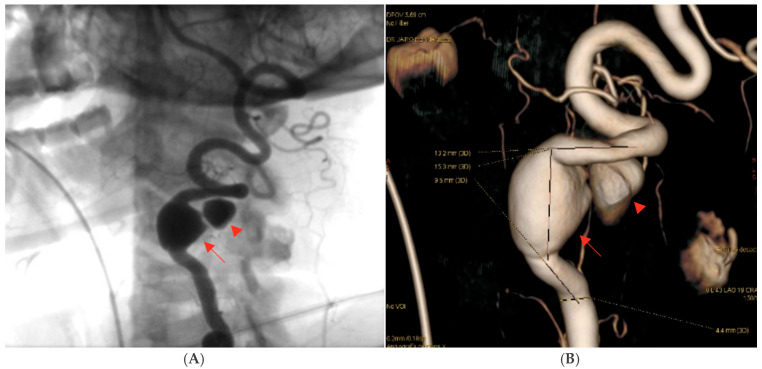
Digital subtraction angiography (DSA) of the left vertebral artery demonstrating a dissecting fusiform aneurysm at the V1–V2 junction with an associated contained pseudoaneurysm. The fusiform aneurysmal dilation measured approximately 18 × 15 mm; the pseudoaneurysm was not quantified separately. The procedure was performed after intra-arterial administration of 10–15 mL of non-ionic iodinated contrast medium (Iohexol, Omnipaque^®^ 300 mg I/mL) per injection, delivered at an injection rate of approximately 4–6 mL/s, with high temporal resolution acquisitions (2–3 frames/s) during the arterial phase: (**A**) Oblique projection after intra-arterial contrast administration showing the dissecting fusiform aneurysm. (**B**) Three-dimensional reconstruction confirming the aneurysmal morphology and vessel anatomy. The aneurysm is indicated by red arrows, while the mural defect is highlighted by a red arrowhead. The remaining vertebrobasilar segments were unremarkable. Source: patient’s medical record, published with prior authorization.

Digital subtraction angiography (DSA) confirmed the presence of a dissecting fusiform aneurysm of the left vertebral artery at the V1–V2 junction, associated with a contained pseudoaneurysm. The true aneurysmal dilation measured 18 × 15 mm, while the pseudoaneurysm was not quantified separately. The V1 (pre-foraminal), V3 (extradural), V4 (intracranial) segments, and the basilar artery were normal. A right P1 segment agenesis with a fetal-type posterior communicating artery was also observed (Figure 3). The apparent discrepancy in aneurysm size between CTA and DSA can be explained by the different imaging principles: CTA captured both the intraluminal aneurysmal dilation and the adjacent perivascular hematoma/pseudoaneurysmal component, whereas DSA more accurately reflected the true intraluminal dimensions of the dissecting aneurysm.

**Figure 4:**
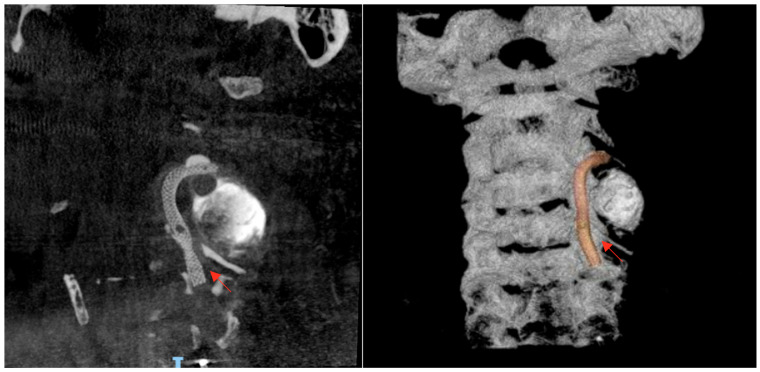
Post-procedural digital subtraction angiography (DSA) and three-dimensional reconstruction images following reconstructive endovascular treatment with a flow-diverting stent (Pipeline Shield). The stent is clearly visualized along the course of the left vertebral artery and is indicated by the red arrow. Source: patient’s medical record, published with prior authorization.

The case was urgently discussed in a multidisciplinary neurovascular team meeting comprising radiologists, interventional neurosurgeons, and neurologists. After a detailed discussion with the patient regarding the risks and benefits of available therapies, it was decided, considering the time of evolution, the patient’s age, and the aneurysm’s characteristics, to perform a reconstructive endovascular treatment using a flow-diverting stent (Pipeline Shield) (Micro Therapeutics Inc., Irvine, CA, USA) (Figure 4), initiating dual antiplatelet therapy (clopidogrel 75 mg plus acetylsalicylic acid 100 mg starting 5 days prior to the procedure and continued for 90 days). The procedure was performed using a triaxial system: a 5 Fr Navien catheter was advanced with the aid of a 0.035 guidewire. Through this, a Phenom 27 microcatheter (Medtronic, Minneapolis, MN, USA) and a 0.014 Synchro microwire (Stryker Neurovascular, Fremont, CA, USA) were navigated across the aneurysmal sac with subtle maneuvers under continuous fluoroscopic and roadmapping guidance. The distal tip of the microcatheter was successfully positioned in the left V3 segment. After systemic intravenous heparinization, the microwire was withdrawn, and a single Pipeline Shield flow-diverting and artery-remodeling stent (4.5 × 35 mm) was advanced and deployed, achieving a healthy-to-healthy proximal artery reconstruction and completely covering the aneurysm neck, which initiated its occlusion process. Control angiograms demonstrated adequate stent apposition, maintained parent vessel patency, and no periprocedural complications. Femoral access was closed with an 8 Fr Angio-Seal device (Terumo Medical Corporation, Somerset, NJ, USA). Post-procedural imaging follow-up with digital subtraction angiography (DSA) confirmed stent positioning and vessel patency.

The patient experienced gradual improvement of neurological symptoms following the procedure, with complete resolution of dysarthria and reduction in vertigo within 48 h. Dual antiplatelet therapy (clopidogrel plus acetylsalicylic acid) was well tolerated, with no hemorrhagic events or clinical complications during hospitalization. She was discharged on day 7 with an NIHSS score of 2 (attributable to bilateral limb ataxia, with improvement mainly in dysarthria) and a modified Rankin Scale (mRS) score [10] of 1, indicating mild symptoms but preserved independence. Telephone follow-up at 30, 90, and 180 days revealed no changes in the mRS score.

## 3. Discussion

Cerebrovascular aneurysms located in intracranial segments account for 97% of cases, most commonly found in the carotid system. The remaining 3% originate in the vertebrobasilar region. Vertebral artery aneurysms are rare, and those located in extracranial segments are even more uncommon [1]. These aneurysms often appear following trauma or as pseudoaneurysms secondary to dissections, while primary aneurysms are typically associated with connective tissue anomalies or other genetic disorders. The most frequently affected segment is V3, followed by V1 [11,12,13].

Vertebral artery pathologies such as atherosclerosis, trauma, and dissection account for the majority of strokes originating from the vertebrobasilar system, which represents approximately 25% of all stroke cases [13]. Therefore, a thromboembolic stroke caused by a primary extracranial vertebral artery aneurysm is an extremely unusual finding.

The ischemic events observed in our patient are consistent with the mechanisms described in vertebral artery dissections. These lesions cause cerebral ischemia primarily through embolism secondary to mural thrombosis, and less frequently through hemodynamic compromise due to stenosis or pseudoaneurysm formation [14]. Population-based studies suggest that vertebral artery dissections account for up to 2.6% of cryptogenic strokes in the posterior circulation, underscoring their underrecognized role in young patients [15]. Pathologically, the dissecting process begins with an intramural hematoma or an intimal–medial tear, creating a false lumen that promotes thrombus formation and distal embolization, explaining the occurrence of multifocal infarcts in the vertebrobasilar territory [14,15].

A literature review was conducted to identify published cases of EVAA that led to stroke. Most reported cases correspond to incidental findings or symptoms localized to the neck and thoracic region. Rupture of these aneurysms can present as cervical hematoma and neck pain, and cases of death due to hemorrhage into the thoracic cavity have been reported. Progressive aneurysm growth without rupture may also cause mass-effect symptoms [4,12,16,17].

The natural history of this condition remains poorly understood, which explains the variability in management strategies [13]. In some reports, patients remain stable under observation, while in others, the natural course includes aneurysm enlargement, symptom progression, ischemia (as in our case), or death [18].

Several therapeutic modalities have been proposed for the management of extracranial vertebral artery aneurysms (EVAA), encompassing both open surgical and endovascular strategies. Conventional surgical approaches include simple aneurysm ligation, a technique aimed at excluding the aneurysmal sac from the circulation. Although effective in selected saccular aneurysms, this procedure was not considered suitable in our patient because of the fusiform morphology of the lesion, which precludes complete isolation and carries the additional risk of compromising distal vertebrobasilar flow. More sophisticated surgical methods, such as distal vertebral artery revascularization through bypass grafting, have been described as alternatives for preserving blood supply to the posterior circulation. Despite their potential benefit, these procedures are technically demanding and associated with considerable morbidity: reported series indicate a perioperative risk of stroke or death of approximately 3–4%, graft thrombosis rates as high as 8%, and a 2% incidence of spinal cord ischemic injury resulting from interruption of critical radiculomedullary arteries [19]. These risks are particularly concerning in young patients with otherwise favorable prognoses, in whom procedural complications may lead to substantial long-term disability.

Endovascular therapies, increasingly favored over the past two decades, offer a less invasive option. Classical techniques include parent artery occlusion with detachable balloons or coil embolization, both of which have been reported with acceptable efficacy in selected cases [20]. The fundamental goal of these procedures is to exclude the aneurysmal lumen from the circulation while maintaining adequate collateral perfusion of the posterior fossa. However, their utility is mainly restricted to saccular aneurysms with a narrow neck or to lesions located in anatomically favorable segments. Fusiform aneurysms, such as the one observed in our patient, involve a circumferential segment of the arterial wall, making secure packing or occlusion technically challenging and increasing the likelihood of incomplete exclusion or recurrence. Furthermore, parent artery sacrifice is rarely acceptable in the vertebral artery without prior demonstration of sufficient collateral flow, given the risk of ischemia in the brainstem and cerebellum.

In light of these limitations, flow-diverting stents have emerged as a reconstructive alternative. These devices allow vessel wall reconstruction while preserving parent artery patency. Importantly, the majority of large published series correspond to intracranial aneurysms, and only isolated case reports and small series describe extracranial applications. Therefore, extrapolation of intracranial outcomes to extracranial lesions must be cautious. For instance, two extracranial pseudoaneurysms successfully treated with flow-diverters have been reported, supporting their potential safety and efficacy in selected patients [21].

By contrast, larger retrospective series and systematic reviews predominantly focused on intracranial posterior circulation aneurysms have shown encouraging results. A multicenter study of 252 posterior circulation aneurysms treated with flow-diverters (Pipeline or Tubridge) reported complete occlusion in 79.1%, favorable clinical outcomes in 91.0%, and major perioperative complications in 7.5% [22]. Another systematic review found that flow-diverters may carry higher procedure-related complication rates but overall comparable safety, with greater long-term efficacy compared to conventional endovascular methods [23]. These results reinforce the reconstructive potential of flow-diverters, though evidence for extracranial vertebral artery aneurysms remains scarce.

The main limitation of the current evidence is that it is derived almost exclusively from case reports and small retrospective series, which are subject to publication and selection bias. While these reports demonstrate technical feasibility, they provide only low-level evidence, insufficient for guideline development [12,20]. The largest meta-analyses of posterior circulation aneurysms remain retrospective and heterogeneous, often combining different devices and inclusion criteria, which complicates extrapolation to clinical practice [22,23]. Furthermore, the rapid evolution of device technology introduces variability across published cohorts [23]. To establish strong treatment recommendations, prospective multicenter trials comparing flow-diverters with open surgery, traditional embolization, or medical therapy are urgently needed. Additionally, the long-term risks of in-stent stenosis, delayed ischemia, or branch coverage in extracranial vertebral aneurysms remain poorly defined.

This case emphasizes the importance of maintaining a high index of diagnostic suspicion when evaluating vertigo in young patients, particularly when accompanied by neurological signs suggestive of posterior circulation ischemia. Unlike elderly patients, in whom atherothrombotic causes are predominant, uncommon etiologies such as arterial dissections, vascular malformations, or extracranial aneurysms should be systematically considered in younger individuals. In our patient, the diagnosis of an extracranial vertebral artery dissecting aneurysm was only established after advanced imaging with computed tomography angiography (CTA) and digital subtraction angiography (DSA), underscoring the indispensable role of multimodal evaluation in these contexts.

From a therapeutic perspective, flow-diverter stenting provided aneurysm exclusion while preserving vertebrobasilar circulation, resulting in favorable neurological recovery. Our case, together with limited extracranial reports and the broader intracranial experience, suggests that flow-diverters are a feasible option in selected EVAA. Nevertheless, higher-quality prospective evidence is required to determine their long-term safety and efficacy in this setting.

Finally, this report reinforces the relevance of a multidisciplinary approach, with close collaboration between neurology, interventional neuroradiology, and vascular surgery, to optimize clinical decision-making and outcomes. It also stresses the need to expand diagnostic algorithms in young stroke patients to include rare but treatable causes. The early incorporation of advanced imaging modalities such as CTA, DSA, and MR angiography in the evaluation of unexplained vertigo or brainstem symptoms may facilitate prompt diagnosis and management. Recognizing and treating these atypical etiologies not only prevents recurrence but also improves functional recovery and contributes to refining therapeutic strategies in vertebrobasilar stroke.

## 4. Conclusions

Extracranial vertebral artery aneurysms remain an exceptionally rare yet potentially treatable cause of posterior circulation stroke, particularly in young patients without traditional vascular risk factors. This case underscores the value of timely and comprehensive diagnostic evaluation through advanced vascular imaging techniques, which enabled successful endovascular management with a flow-diverting stent as a technically feasible option in selected cases. Nevertheless, current evidence is limited to case reports and small series, highlighting the pressing need for prospective studies. Beyond the individual outcome, this report emphasizes the importance of systematic data collection, multicenter collaboration, and the establishment of dedicated registries to better define the natural history and therapeutic strategies of EVAAs. Developing standardized diagnostic and management protocols will be essential to guide clinical decision-making, optimize patient safety, and strengthen the limited evidence base available for this uncommon yet clinically relevant condition. Finally, it reinforces the role of a multidisciplinary approach and the need to consider rare etiologies in the evaluation of young stroke patients.

## Data Availability

Data is contained within the article.

## References

[1] Morasch M.D., Phade S.V., Naughton P., Garcia-Toca M., Escobar G., Berguer R. (2013). Primary Extracranial Vertebral Artery Aneurysms. Ann. Vasc. Surg..

[2] Strambo D., Peruzzotti-Jametti L., Semerano A., Fanelli G., Simionato F., Chiesa R., Rinaldi E., Martinelli V., Comi G., Bacigaluppi M. (2017). Treatment Challenges of a Primary Vertebral Artery Aneurysm Causing Recurrent Ischemic Strokes. Case Rep. Neurol. Med..

[3] Rai P., Bhattacharya K., Rastogi S., Joshi P., Rabade K., Shetty N., Kulkarni S. (2024). Beyond the Throat: Imaging of Parapharyngeal Space Lesions. Clin. Radiol..

[4] Evans K., Lindert R.-B., Dyde R., Tse G.H. (2022). Chronic Fusiform Extracranial Vertebral Artery Aneurysm with Recurrent Posterior Circulation Emboli: Case Report and Review of the Literature. Interv. Neuroradiol..

[5] Stavrinou L.C., Stranjalis G., Stavrinou P.C., Bontozoglou N., Sakas D.E. (2010). Extracranial Vertebral Artery Aneurysm Presenting as a Chronic Cervical Mass Lesion. Case Rep. Med..

[6] Yang L., Ran H. (2018). Extracranial Vertebral Artery Dissection: Findings and Advantages of Ultrasonography. Medicine.

[7] Provenzale J.M., Sarikaya B. (2009). Comparison of Test Performance Characteristics of MRI, MR Angiography, and CT Angiography in the Diagnosis of Carotid and Vertebral Artery Dissection: A Review of the Medical Literature. Am. J. Roentgenol..

[8] Teasdale G., Maas A., Lecky F., Manley G., Stocchetti N., Murray G. (2014). The Glasgow Coma Scale at 40 Years: Standing the Test of Time. Lancet Neurol..

[9] Alemseged F., Rocco A., Arba F., Schwabova J.P., Wu T., Cavicchia L., Ng F., Ng J.L., Zhao H., Williams C. (2022). Posterior National Institutes of Health Stroke Scale Improves Prognostic Accuracy in Posterior Circulation Stroke. Stroke.

[10] Banks J.L., Marotta C.A. (2007). Outcomes Validity and Reliability of the Modified Rankin Scale: Implications for Stroke Clinical Trials: A Literature Review and Synthesis. Stroke.

[11] Kulubya E.S., Yu N., Castillo J.A., Duong H. (2023). External Carotid Artery-Radial Artery-Vertebral Artery Bypass for Surgical Treatment of Radiculopathy Caused by an Extracranial Vertebral Artery Aneurysm: A Case Report and Review of the Literature. Surg. Neurol. Int..

[12] Pataki Á., Nguyen D.T., Nagy Z., Nardai S., Nemes B. (2022). Stent-Graft Treatment of a Giant Asymptomatic Extracranial Vertebral Artery Aneurysm: Case Report and Literature Review. Ann. Vasc. Surg..

[13] Wang Y., Jiao L. (2021). Endovascular Treatment of a Primary Extracranial Vertebral Artery Aneurysm Causing Ischemic Stroke. Neurol. India.

[14] Schievink W.I. (2001). Spontaneous Dissection of the Carotid and Vertebral Arteries. N. Engl. J. Med..

[15] Debette S., Leys D. (2009). Cervical-Artery Dissections: Predisposing Factors, Diagnosis, and Outcome. Lancet Neurol..

[16] Nenadic D., Balevic M., Milojevic M., Tanskovic S. (2018). Extracranial Vertebral Artery Aneurysm Rupture Complicated by Extrapleural Haematoma. EJVES Short Rep..

[17] Rifkinson-Mann S., Laub J., Haimov M. (1986). Atraumatic Extracranial Vertebral Artery Aneurysm: Case Report and Review of the Literature. J. Vasc. Surg..

[18] Kim H.S., Choi C.H., Lee T.H., Kim S.P. (2010). Fusiform Aneurysm Presenting with Cervical Radiculopathy in Ehlers-Danlos Syndrome. J. Korean Neurosurg. Soc..

[19] Takahashi S., Katayama K., Tatsugawa T., Sueda T. (2015). A Successful Hybrid Repair for Vertebral Arteriovenous Fistula with Extracranial Vertebral Artery Aneurysm. Ann. Vasc. Surg..

[20] Cohen J.E., Gomori J.M., Rajz G., Rosenthal G., El Hassan H.A., Moscovici S., Itshayek E. (2016). Vertebral Artery Pseudoaneurysms Secondary to Blunt Trauma: Endovascular Management by Means of Neurostents and Flow Diverters. J. Clin. Neurosci..

[21] Plou P., Landriel F., Beltrame S., Hem S., Peralta O., García-Mónaco R., Yampolsky C. (2019). Flow Diverter for the Treatment of Pseudoaneurysms of the Extracraneal Vertebral Artery: Report of Two Cases and Review of the Literature. World Neurosurg..

[22] Qi P., Tong X., Liang X., Xue X., Wu Z., Feng X., Zhang M., Jiang Z., Wang D., Liu A. (2023). Flow Diversion for Posterior Circulation Aneurysms: A Multicenter Retrospective Study. Ther. Adv. Neurol. Disord..

[23] Li S., Zeng C., Tao W., Huang Z., Yan L., Tian X., Chen F. (2022). The Safety and Efficacy of Flow Diversion versus Conventional Endovascular Treatment for Intracranial Aneurysms: A Meta-Analysis of Real-World Cohort Studies from the Past 10 Years. Am. J. Neuroradiol..

